# Supplementary material for: Beyond Histones: Unveiling the Functional Roles of Protein Acetylation in Prokaryotes and Eukaryotes
Source: Cell Biol Int. 2025 Jul 5;49(10):1301–18. doi: 10.1002/cbin.70055 (PMC12445822; doi:10.1002/cbin.70055)
Supplement: Supplementary file 1 — Supplementary Table 1. [file CBIN-49-1301-s001.docx]

Supplementary Table 1. List of selected acetylomes in this study.

| **Group** | **Specie** | **Number of Kac sites** | **Number of Kac proteins** | **REF** |
| --- | --- | --- | --- | --- |
| Archaea | *Haloferax mediterranei* | 1017 | 643 | (1) |
| Bacteria | *Bacillus subtilis* | 1355 | 629 | (2) |
| Bacteria | *Escherichia coli* | 2502 | 809 | (3) |
| Bacteria | *Mycobacterium tuberculosis* | 1128 | 658 | (4) |
| Bacteria | *Pseudomonas aeruginosa* | 1102 | 522 | (5) |
| Bacteria | *Thermus thermophilus* | 197 | 124 | (6) |
| Bacteria | *Vibrio cholerae* | 3402 | 1240 | (7) |
| Fungi | *Aspergillus flavus* | 1383 | 652 | (8) |
| Fungi | *Aspergillus fumigatus* | 5328 | 2312 | (9) |
| Fungi | *Beauveria bassiana* | 3421 | 1458 | (10) |
| Fungi | *Botrytis cinerea* | 1582 | 954 | (11) |
| Fungi | *Candida albicans* | 2048 | 926 | (9) |
| Fungi | *Cryptococcus neoformans* | 3535 | 1461 | (9) |
| Fungi | *Fusarium graminearium* | 517 | 364 | (12) |
| Fungi | *Histoplasma capsulatum* | 775 | 456 | (13) |
| Fungi | *Magnaporthe oryzae_hyphae* | 2720 | 1269 | (14) |
| Fungi | *Magnaporthe oryzae_mycelia* | 1551 | 704 | (15) |
| Fungi | *Phytophthora sojae* | 2197 | 1150 | (16) |
| Fungi | *Saccharomyces cerevisiae* | 4120 | 1321 | (17) |
| Fungi | *Trichophyton rubrum*_conidia | 386 | 285 | (18) |
| Fungi | Trichophyton rubrum_mycelia | 5414 | 2335 | (18) |
| Fungi | *Yarrowia lipolytica* | 3163 | 1428 | (19) |
| Protozoan | *Plasmodium falciparum* | 2876 | 1146 | (20) |
| Protozoan | *Toxoplasma gondii (*extra) | 571 | 386 | (21) |
| Protozoan | *Toxoplasma gondii* (intra) | 411 | 274 | (22) |
| Protozoan | *Trypanosoma brucei (*blood) | 7445 | 2533 | (23) |
| Protozoan | *Trypanosoma brucei* (procyclic) | 288 | 210 | (24) |
| Protozoan | *Trypanosoma evansi* | 4965 | 1942 | (23) |
| Protozoan | *Trypanosoma cruzi* | 389 | 235 | (24) |
| Protozoan | *Giardia lamblia* | 2999 | 956 | (25) |
| Worms | *Schistosoma japonicum* (adult) | 2393 | 1109 | (26) |
| Worms | *Schistosoma japonicum* (juvenile male) | 102 | 71 | (27) |
| Worms | *Schistosoma japonicum* (juvenile female) | 231 | 162 | (27) |
| Worms | *Schistosoma japonicum* (adult male) | 616 | 346 | (27) |
| Worms | *Schistosoma japonicum* (adult female) | 430 | 281 | (27) |
| Worms | *Trichinella spiralis* | 3872 | 1592 | (28) |
| Plants | *Arabidopsis thaliana* | 2152 | 1022 | (29) |
| Plants | *Brachypodium distachyon* | 636 | 353 | (30) |
| Plants | *Fragaria* sp | 1392 | 684 | (31) |
| Plants | *Oryza sativa* | 1353 | 866 | (32) |
| Plants | *Triticum aestivum* | 416 | 277 | (33) |
| Insects | *Bombyx mori* | 667 | 342 | (34) |
| Insects | *Drosophila melanogaster* | 1981 | 1013 | (35) |
| Fish | *Danio rerio* | 377 | 189 | (36) |
| Mammals | HeLA cells | 3345 | 1440 | (37) |
| Mammals | U2OS cells | 3174 | 855 | (38) |
| Mammals | Human skeletal muscle tissue | 2811 | 991 | (39) |
| Mammals | *Rattus norvegicus* (16 different tissues) | 15474 | 4541 | (39) |

References

1. Liu J, Wang Q, Jiang X, Yang H, Zhao D, Han J, et al. Systematic Analysis of Lysine Acetylation in the Halophilic Archaeon Haloferax mediterranei. J Proteome Res. 2017;16(9).

2. Kosono S, Tamura M, Suzuki S, Kawamura Y, Yoshida A, Nishiyama M, et al. Changes in the acetylome and succinylome of Bacillus subtilis in response to carbon source. PLoS One. 2015;10(6).

3. Castaño‐Cerezo S, Bernal V, Post H, Fuhrer T, Cappadona S, Sánchez‐Díaz NC, et al. Protein acetylation affects acetate metabolism, motility and acid stress response in Escherichia coli . Mol Syst Biol. 2014;10(11).

4. Xie L, Wang X, Zeng J, Zhou M, Duan X, Li Q, et al. Proteome-wide lysine acetylation profiling of the human pathogen Mycobacterium tuberculosis. International Journal of Biochemistry and Cell Biology. 2015;59.

5. Gaviard C, Broutin I, Cosette P, Dé E, Jouenne T, Hardouin J. Lysine Succinylation and Acetylation in Pseudomonas aeruginosa. J Proteome Res. 2018;17(7).

6. Okanishi H, Kim K, Masui R, Kuramitsu S. Acetylome with structural mapping reveals the significance of lysine acetylation in Thermus thermophilus. J Proteome Res. 2013;12(9).

7. Jers C, Ravikumar V, Lezyk M, Sultan A, Sjöling Å, Wai SN, et al. The global acetylome of the human pathogen vibrio cholerae V52 reveals lysine acetylation of major transcriptional regulators. Front Cell Infect Microbiol. 2018;7(JAN).

8. Lv Y. Proteome-wide profiling of protein lysine acetylation in Aspergillus flavus. PLoS One. 2017;12(6).

9. Li Y, Li H, Sui M, Li M, Wang J, Meng Y, et al. Fungal acetylome comparative analysis identifies an essential role of acetylation in human fungal pathogen virulence. Commun Biol. 2019;2(1).

10. Cai Q, Tian L, Xie JT, Huang QY, Feng MG, Keyhani NO. A fungal sirtuin modulates development and virulence in the insect pathogen, Beauveria bassiana. Environ Microbiol. 2021;23(9).

11. Lv B, Yang Q, Li D, Liang W, Song L. Proteome-wide analysis of lysine acetylation in the plant pathogen Botrytis cinerea. Sci Rep. 2016;6.

12. Zhou S, Yang Q, Yin C, Liu L, Liang W. Systematic analysis of the lysine acetylome in Fusarium graminearum. BMC Genomics. 2016;17(1).

13. Xie L, Fang W, Deng W, Yu Z, Li J, Chen M, et al. Global profiling of lysine acetylation in human histoplasmosis pathogen Histoplasma capsulatum. International Journal of Biochemistry and Cell Biology. 2016;73.

14. Sun X, Li Z, Liu H, Yang J, Liang W, Peng YL, et al. Large-scale identification of lysine acetylated proteins in vegetative hyphae of the rice blast fungus. Sci Rep. 2017;7(1).

15. Liang M, Zhang S, Dong L, Kou Y, Lin C, Dai W, et al. Label-Free Quantitative Proteomics of Lysine Acetylome Identifies Substrates of Gcn5 in Magnaporthe oryzae Autophagy and Epigenetic Regulation. mSystems. 2018;3(6).

16. Li D, Lv B, Tan L, Yang Q, Liang W. Acetylome analysis reveals the involvement of lysine acetylation in diverse biological processes in Phytophthora sojae. Sci Rep. 2016;6.

17. Weinert BT, Iesmantavicius V, Moustafa T, Schölz C, Wagner SA, Magnes C, et al. Acetylation dynamics and stoichiometry in Saccharomyces cerevisiae. Mol Syst Biol. 2014;10(1).

18. Xu X, Liu T, Yang J, Chen L, Liu B, Wang L, et al. The First Whole-Cell Proteome- and Lysine-Acetylome-Based Comparison between Trichophyton rubrum Conidial and Mycelial Stages. J Proteome Res. 2018;17(4).

19. Wang G, Guo L, Liang W, Chi Z, Liu L. Systematic analysis of the lysine acetylome reveals diverse functions of lysine acetylation in the oleaginous yeast Yarrowia lipolytica. AMB Express. 2017;7(1).

20. Cobbold SA, Santos JM, Ochoa A, Perlman DH, Llinas M. Proteome-wide analysis reveals widespread lysine acetylation of major protein complexes in the malaria parasite. Sci Rep. 2016;6.

21. Xue B, Jeffers V, Sullivan WJ, Uversky VN. Protein intrinsic disorder in the acetylome of intracellular and extracellular Toxoplasma gondii. Mol Biosyst. 2013;9(4).

22. Jeffers V, Sullivan WJ. Lysine acetylation is widespread on proteins of diverse function and localization in the protozoan parasite Toxoplasma gondii. Eukaryot Cell. 2012;11(6).

23. Zhang N, Jiang N, Zhang K, Zheng L, Zhang D, Sang X, et al. Landscapes of Protein Posttranslational Modifications of African Trypanosoma Parasites. iScience. 2020;23(5).

24. Moretti NS, Cestari I, Anupama A, Stuart K, Schenkman S. Comparative Proteomic Analysis of Lysine Acetylation in Trypanosomes. J Proteome Res. 2018;17(1).

25. Zhu W, Jiang X, Sun H, Li Y, Shi W, Zheng M, et al. Global lysine acetylation and 2-hydroxyisobutyrylation profiling reveals the metabolism conversion mechanism in giardia lamblia. Molecular and Cellular Proteomics. 2021;20.

26. Hong Y, Cao X, Han Q, Yuan C, Zhang M, Han Y, et al. Proteome-wide analysis of lysine acetylation in adult Schistosoma japonicum worm. J Proteomics. 2016;148.

27. Li Q, Zhao N, Liu M, Shen H, Huang L, Mo X, et al. Comparative analysis of proteome-wide lysine acetylation in juvenile and adult Schistosoma japonicum. Front Microbiol. 2017;8(NOV).

28. Yang Y, Tong M, Bai X, Liu X, Cai X, Luo X, et al. Comprehensive proteomic analysis of lysine acetylation in the Foodborne pathogen Trichinella spiralis. Front Microbiol. 2018;8(JAN).

29. Hartl M, Füßl M, Boersema PJ, Jost J, Kramer K, Bakirbas A, et al. Lysine acetylome profiling uncovers novel histone deacetylase substrate proteins in Arabidopsis . Mol Syst Biol. 2017;13(10).

30. Zhen S, Deng X, Wang J, Zhu G, Cao H, Yuan L, et al. First Comprehensive Proteome Analyses of Lysine Acetylation and Succinylation in Seedling Leaves of Brachypodium distachyon L. Sci Rep. 2016;6.

31. Fang X, Chen W, Zhao Y, Ruan S, Zhang H, Yan C, et al. Global analysis of lysine acetylation in strawberry leaves. Front Plant Sci. 2015;6(September).

32. Xue C, Liu S, Chen C, Zhu J, Yang X, Zhou Y, et al. Global Proteome Analysis Links Lysine Acetylation to Diverse Functions in Oryza Sativa. Proteomics. 2018;18(1).

33. Zhang Y, Song L, Liang W, Mu P, Wang S, Lin Q. Comprehensive profiling of lysine acetylproteome analysis reveals diverse functions of lysine acetylation in common wheat. Sci Rep. 2016;6.

34. Nie Z, Zhu H, Zhou Y, Wu C, Liu Y, Sheng Q, et al. Comprehensive profiling of lysine acetylation suggests the widespread function is regulated by protein acetylation in the silkworm, Bombyx mori. Proteomics. 2015;15(18).

35. Weinert BT, Wagner SA, Horn H, Henriksen P, Liu WR, Olsen J V., et al. Proteome-wide mapping of the Drosophila acetylome demonstrates a high degree of conservation of lysine acetylation. Sci Signal. 2011;4(183).

36. Kwon OK, Kim S, Lee S. Global proteomic analysis of lysine acetylation in zebrafish (Danio rerio) embryos. Electrophoresis. 2016;37(23–24).

37. Xu H, Chen X, Xu X, Shi R, Suo S, Cheng K, et al. Lysine Acetylation and Succinylation in HeLa Cells and their Essential Roles in Response to UV-induced Stress. Sci Rep. 2016;6.

38. Sol EM, Wagner SA, Weinert BT, Kumar A, Kim HS, Deng CX, et al. Proteomic Investigations of Lysine Acetylation Identify Diverse Substrates of Mitochondrial Deacetylase Sirt3. PLoS One. 2012;7(12).

39. Lundby A, Lage K, Weinert BT, Bekker-Jensen DB, Secher A, Skovgaard T, et al. Proteomic Analysis of Lysine Acetylation Sites in Rat Tissues Reveals Organ Specificity and Subcellular Patterns. Cell Rep. 2012;2(2).
