# Supplementary material for: Beyond Histones: Unveiling the Functional Roles of Protein Acetylation in Prokaryotes and Eukaryotes
Source: Cell Biol Int. 2025 Jul 5;49(10):1301–18. doi: 10.1002/cbin.70055 (PMC12445822; doi:10.1002/cbin.70055)
Supplement: Supplementary file 2 — Supmat. [file CBIN-49-1301-s002.docx]

Supplementary Figures


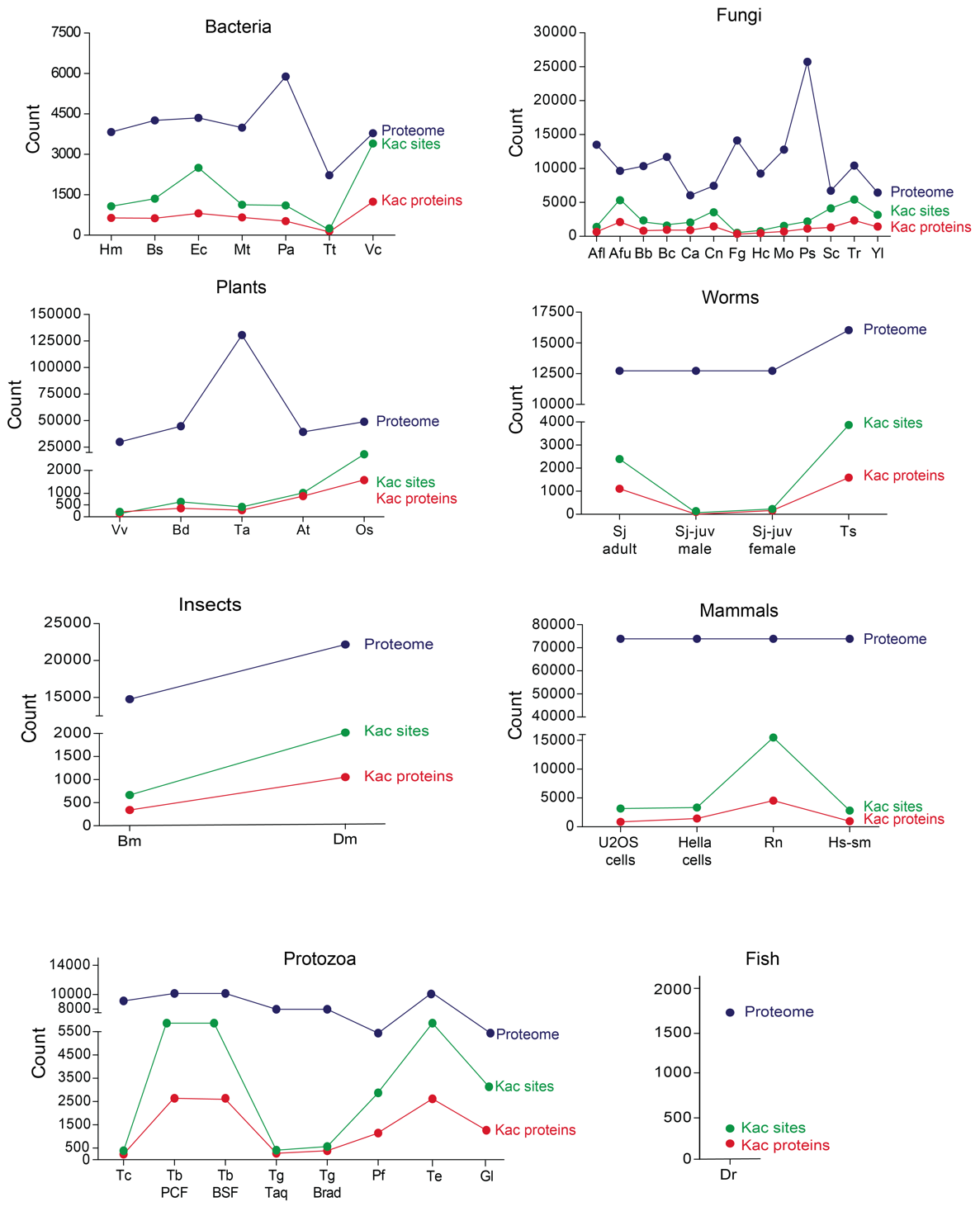


**Figure S1. Distribution of acetylomes selected for this work based on proteome size, number of acetylated sizes and acetylated proteins.** The acetylome descriptions of all species for each group of organisms (bacteria, fungi, plants, worms, insects, mammals and protozoa) were plotted to give a broad idea about the characteristics of each acetylome. Each plot contains the size of the proteome, the number of acetylated-lysine sites (Kac sites) and acetylated proteins (Kac proteins). Hm (*Haloferax mediterranei*); Bs (*Bacillus subtilis*); Ec (*Escherichia coli*); Mt (*Mycobacterium tuberculosis*); Pa (*Pseudomonas aeruginosa*); Tt (*Thermus thermophilus*); Vc (*Vibrio colerae*); Afl (*Aspergillus flavus*); Afu (*Aspergillus fumigatus*); Bb (*Beauveria bassiana*); Bc (*Botrytis cinerea*); Ca (*Candida albicans*); Cn (*Cryptococcus neoformans*); Fg (*Fusarium graminearium*); Hc (*Histoplasma capsulatum*); Mo (*Magnaporthe oryzae*); Ps (*Phytophthora sojae*); Sc (*Saccharomyces cerevisiae*); Tr (*Trichophyton rubrum*); Yl (*Yarrowia lipolytica*); At (*Arabdopsis thaliana*); Os (*Oryza sativa*); Ta (*Triticum aestivum*); Bd (*Brachypodium distachyon*); Vv (*Vitis vinifera*); Sj adult (*Schistosoma japonicum* adult); Sj-juv male (*Schistosoma japonicum* juvenile male form); Sj-juv female (*Schistosoma japonicum* juvenile female form); Ts (*Trichinella spiralis*); Bm (*Bombyx mori*); Dm (*Drosophila melanogaster*); Tc (*Trypanosoma cruzi*); Tg Taq (*Toxoplasma gondii, tachyzoite form*); Tg Brad (*Toxoplasma gondii, bradyzoite form*); Tb PCF (*Trypanosoma brucei* procyclic form); Tb BSD (*Trypanosoma brucei* bloodstream form); Pf (*Plasmodium falciparum*); Te (*Trypanosoma evansi*); Gl (*Giardia lamblia*); Dr (*Danio rerio*); Rn (*Rattus novergicus*); Hs-sm (*Homo sapiens* skeletal muscle).


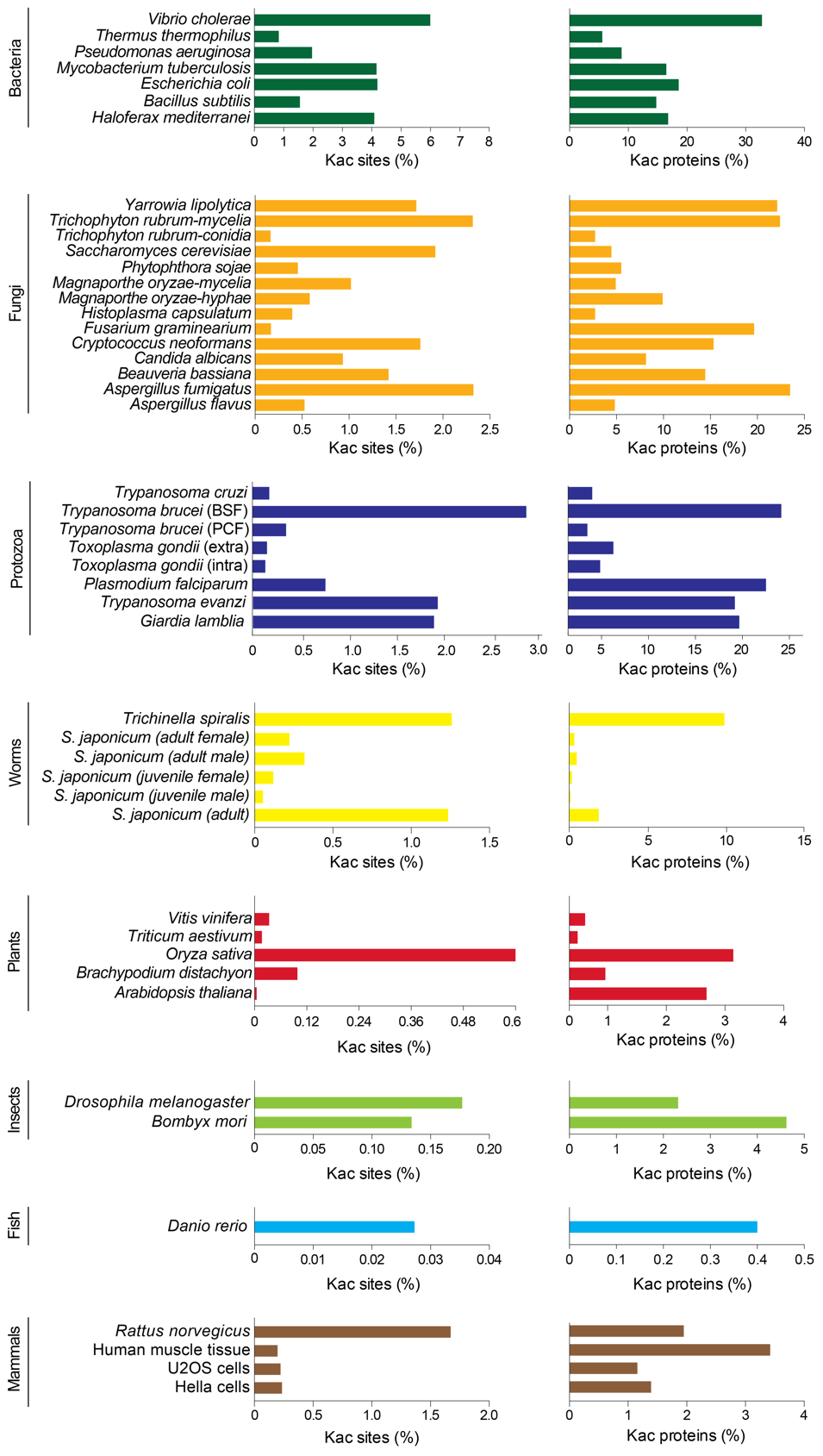


**Figure S2. Percentage of the lysine-acetylated sites and lysine-acetylated proteins found in each group of organisms.** The percentages of the lysine-acetylated sites and lysine-acetylated proteins were calculated based on the total number of lysine residues found in each proteome and the size of the proteome from each specie.


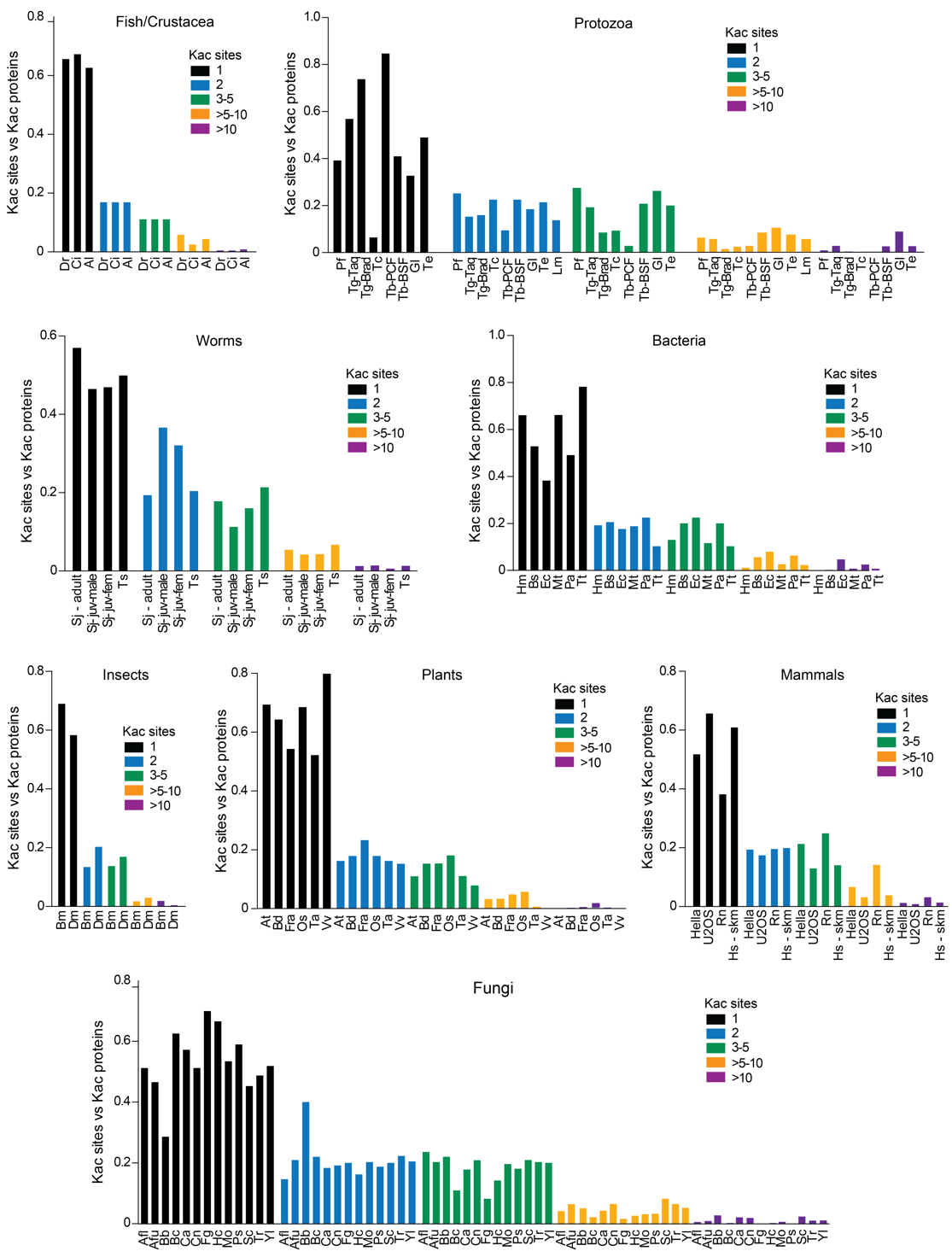


**Figure S3. Number of Kac sites detected in each protein in all acetylomes analyzed.** The number of proteins identified with 1, 2, 3-5, >5-10 and >10 Kac were quantified using the acetylome data for each specie analyzed. Hm (*Haloferax mediterranei*); Bs (*Bacillus subtilis*); Ec (*Escherichia coli*); Mt (*Mycobacterium tuberculosis*); Pa (*Pseudomonas aeruginosa*); Tt (*Thermus thermophilus*); Vc (*Vibrio colerae*); Afl (*Aspergillus flavus*); Afu (*Aspergillus fumigatus*); Bb (*Beauveria bassiana*); Bc (*Botrytis cinerea*); Ca (*Candida albicans*); Cn (*Cryptococcus neoformans*); Fg (*Fusarium graminearium*); Hc (*Histoplasma capsulatum*); Mo (*Magnaporthe oryzae*); Ps (*Phytophthora sojae*); Sc (*Saccharomyces cerevisiae*); Tr (*Trichophyton rubrum*); Yl (*Yarrowia lipolytica*); At (*Arabdopsis thaliana*); Os (*Oryza sativa*); Ta (*Triticum aestivum*); Bd (*Brachypodium distachyon*); Vv (*Vitis vinifera*); Sj adult (*Schistosoma japonicum* adult); Sj-juv male (*Schistosoma japonicum* juvenile male form); Sj-juv female (*Schistosoma japonicum* juvenile female form); Ts (*Trichinella spiralis*); Bm (*Bombyx mori*); Dm (*Drosophila melanogaster*); Tc (*Trypanosoma cruzi*); Tg Taq (*Toxoplasma gondii, tachyzoite form*); Tg Brad (*Toxoplasma gondii, bradyzoite form*); Tb PCF (*Trypanosoma brucei* procyclic form); Tb BSD (*Trypanosoma brucei* bloodstream form); Pf (*Plasmodium falciparum*); Te (*Trypanosoma evansi*); Gl (*Giardia lamblia*); Dr (*Danio rerio*); Rn (*Rattus novergicus*); Hs-sm (*Homo sapiens* skeletal muscle).


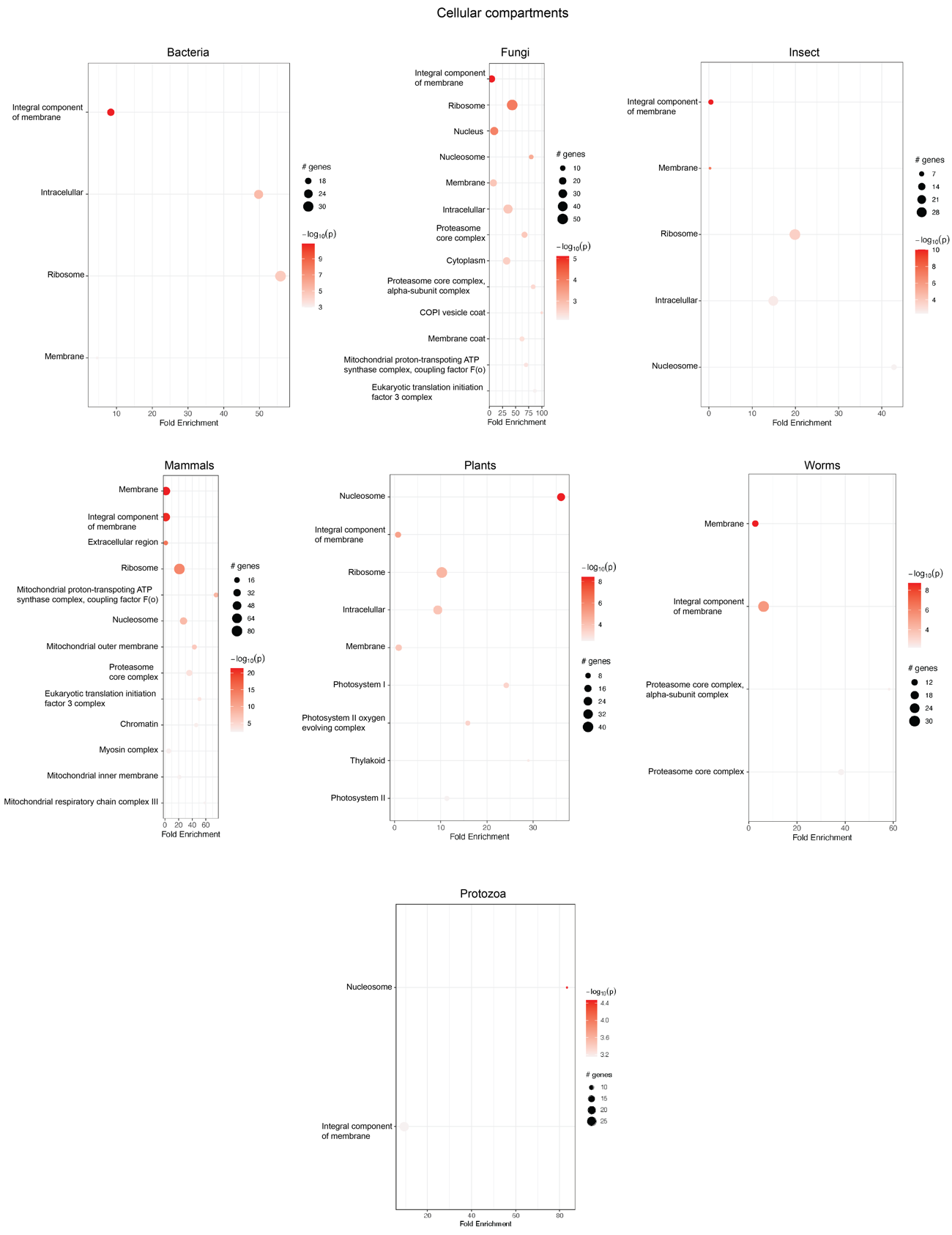


**Figure S4. Cellular component enrichment across analyzed groups.** Here we demonstrated the cellular component categories enriched in at least two different species within each analyzed group.


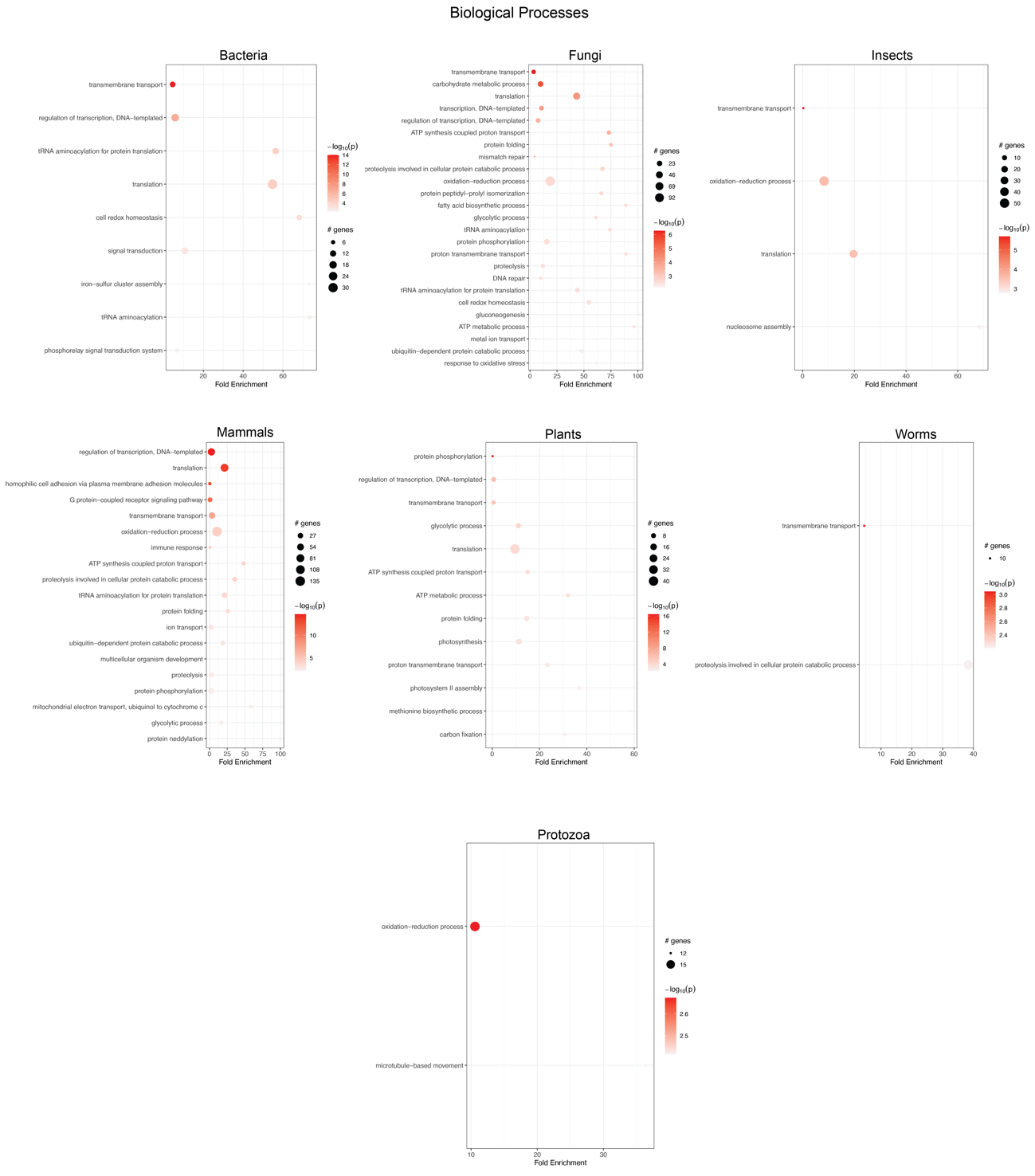


**Figure S5.** **Biological processes enrichment** **across analyzed groups.** Here we demonstrated the biological processes categories enriched in at least two different species within each analyzed group.


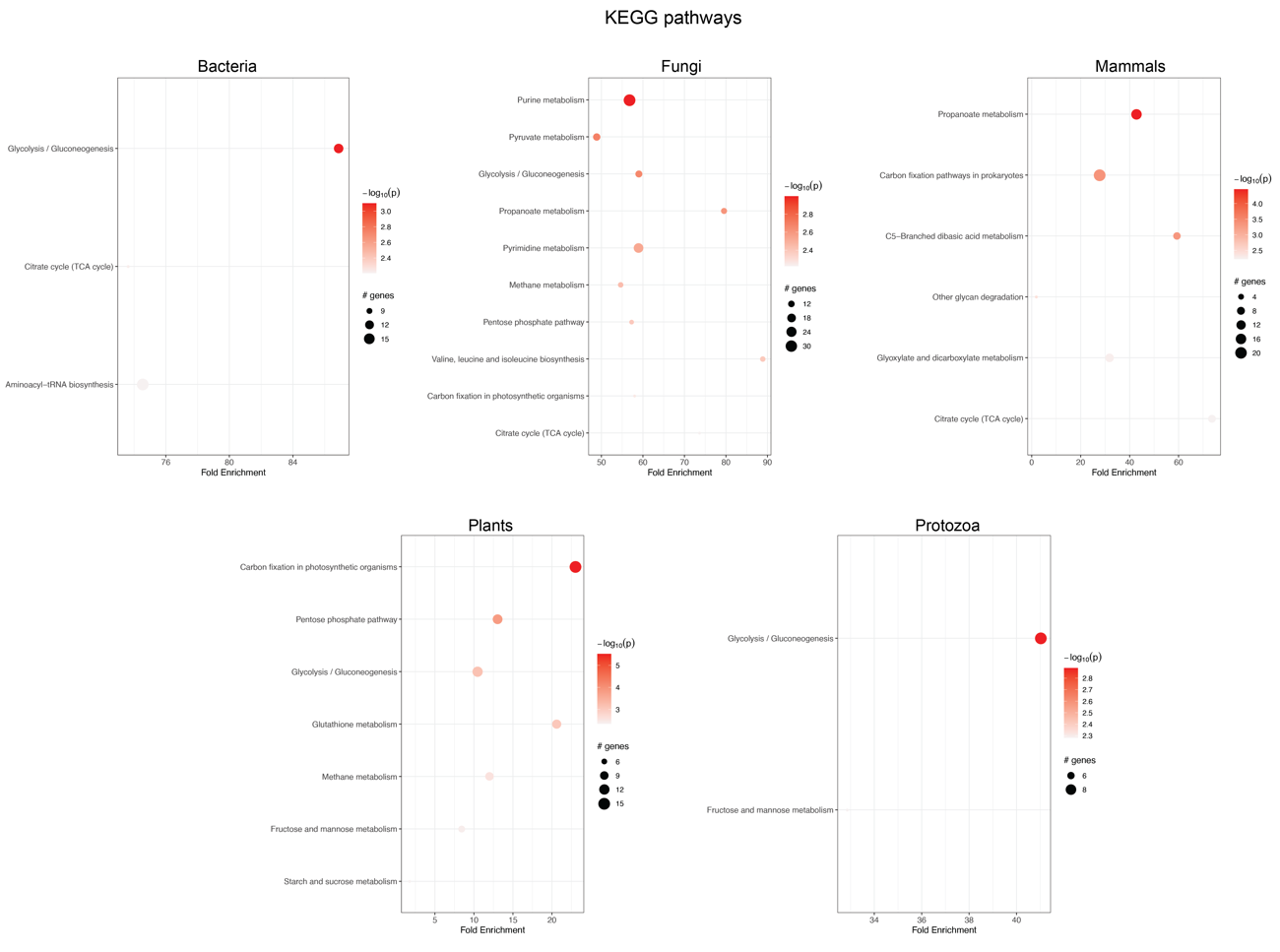


**Figure S6.** **Biological processes enrichment** **across analyzed groups.** Here we demonstrated the KEGG categories enriched in at least two different species within each analyzed group.


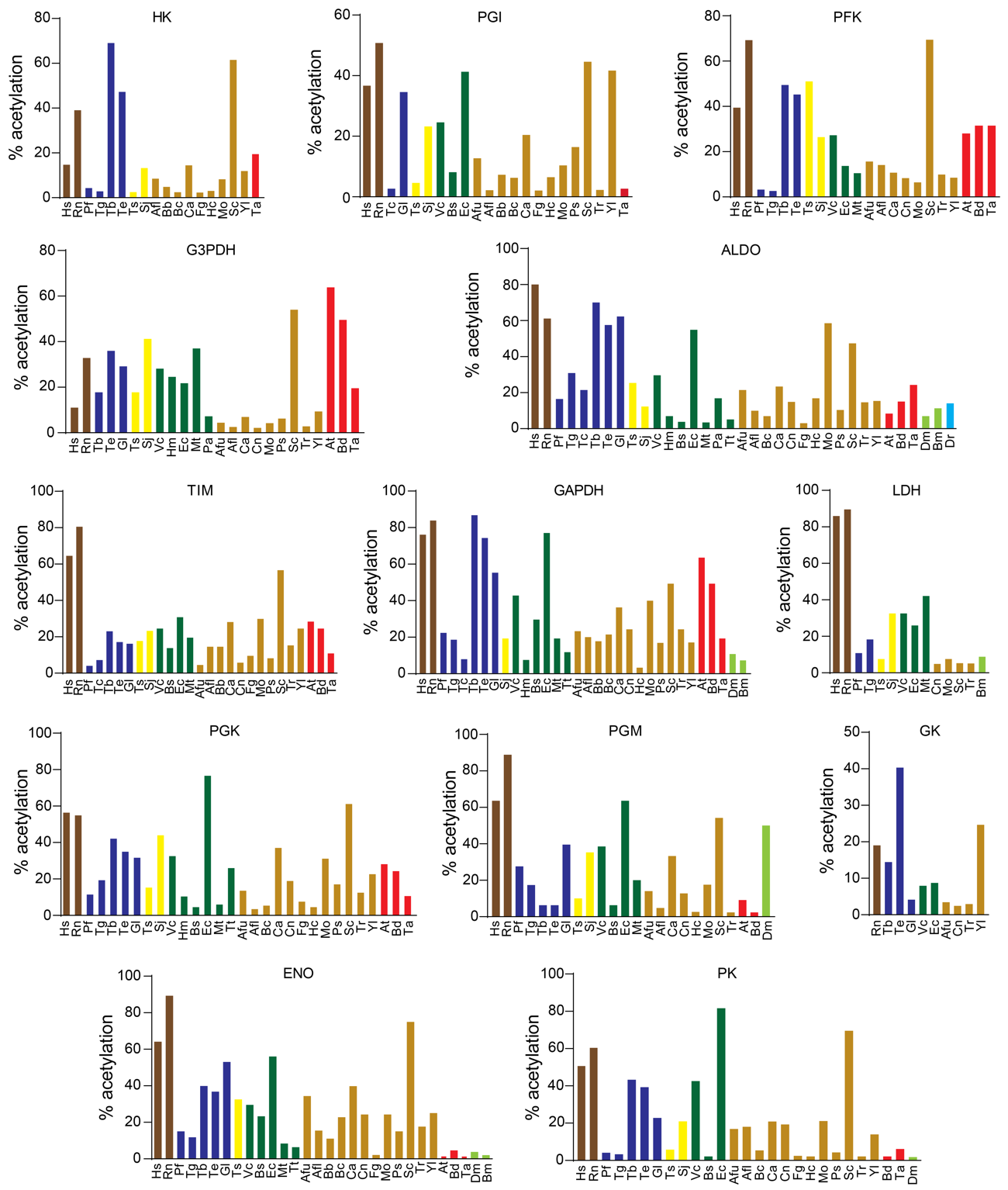


**Figure S7. Glycolytic enzymes percentage of the lysine-acetylated and non-acetylated found in each group of organisms of different species.** Enzymes were considered acetylated even if only one Kac site had been identified in the specific acetylome. Hexokinase (HK), glucose phosphate isomerase (PGI); phosphofructokinase (PFK); fructose-1,6-bisphosphate aldolase (ALD); glyceraldehyde-3-phosphate dehydrogenase (GAPDH), triose-phosphate isomerase (TIM); phosphoglycerate kinase (PGK); phosphoglycerate mutase (PGM); enolase (ENO); pyruvate kinase (PK). The groups are represented by different colors as follows: mammals (brown); protozoan (blue); worms (yellow); bacteria (green); fungi (gold); plants (red); insects (light green); fish (cyan). Identified species: Hs (*Homo sapiens)*; Rn (*Rattus* novergicus); Tb (*trypanosoma* brucei); Tc (*Trypanosoma cruzi*); Tg (*Toxoplasma gondii*); Pf (*Plasmodium falciparum*); Te (*Trypanosoma evansi*); Gl (*Giardia lamblia*); Hm (*Haloferax mediterranei*); Bs (*Bacillus subtilis*); Ec (*Escherichia coli*); Mt (*Mycobacterium tuberculosis*); Pa (*Pseudomonas aeruginosa*); Tt (*Thermus thermophilus*); Vc (*Vibrio colerae*); Afl (*Aspergillus flavus*); Afu (*Aspergillus fumigatus*); Bb (*Beauveria bassiana*); Bc (*Botrytis cinerea*); Ca (*Candida albicans*); Cn (*Cryptococcus neoformans*); Fg (*Fusarium graminearium*); Hc (*Histoplasma capsulatum*); Mo (*Magnaporthe oryzae*); Ps (*Phytophthora sojae*); Sc (*Saccharomyces cerevisiae*); Tr (*Trichophyton rubrum*); Yl (*Yarrowia lipolytica*); At (*Arabdopsis thaliana*); Os (*Oryza sativa*); Ta (*Triticum aestivum*); Bd (*Brachypodium distachyon*); Vv (*Vitis vinifera*); Sj (*Schistosoma japonicum*); Ts (*Trichinella spiralis*); Bm (*Bombyx mori*); Dm (*Drosophila melanogaster*); Dr (*Danio rerio*).


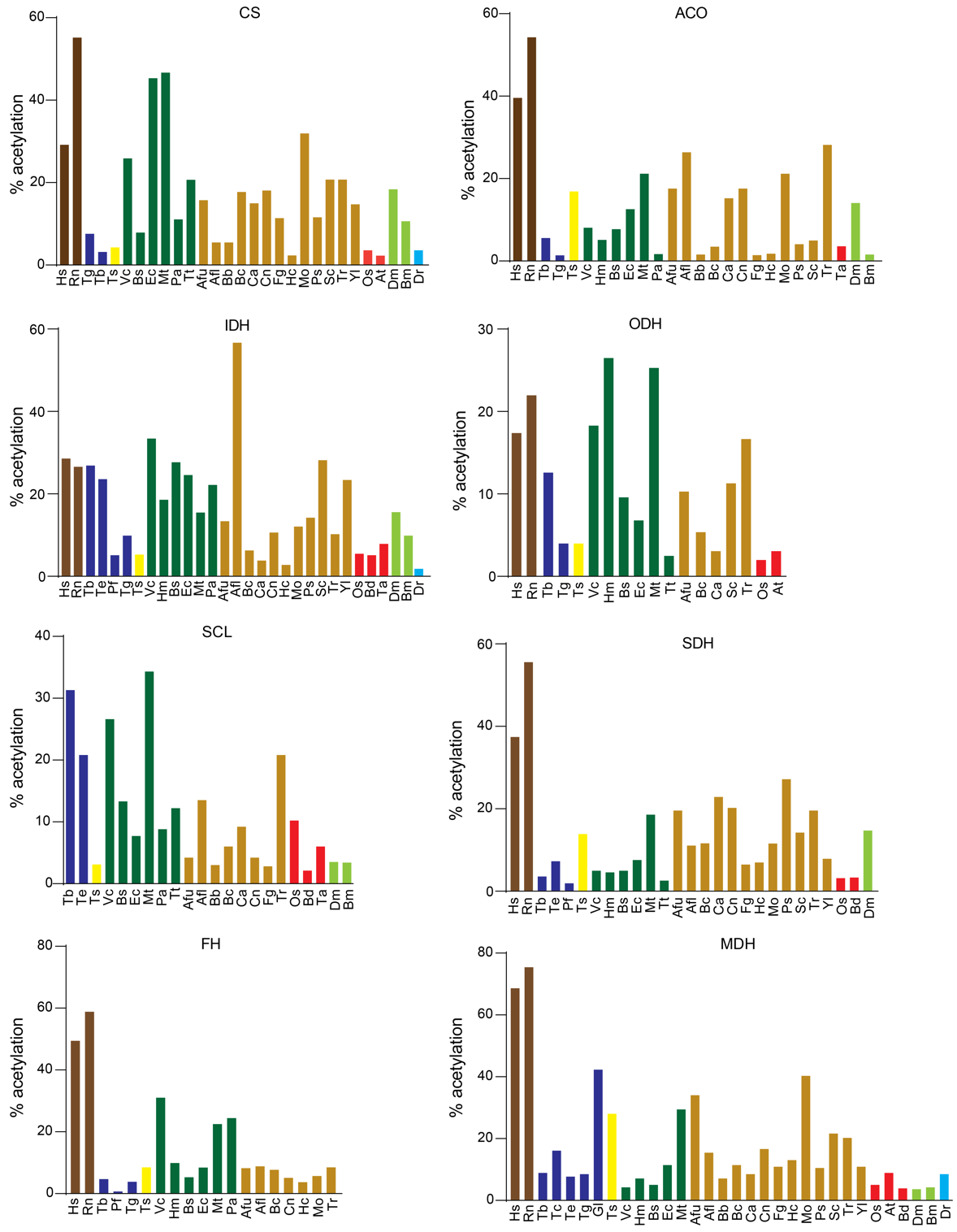


**Figure S8. TCA cycle enzymes percentage of the lysine-acetylated and non-acetylated found in each group of organisms of different species.** Enzymes were considered acetylated even if only one Kac site had been identified in the specific acetylome. Citrate synthase (CS), Aconitate (ACO), Isocitrate dehydrogenase (IDH), α-Ketoglutarate (ODH), Succinyl-CoA synthetase (SCL), Succinate dehydrogenase (SDH), Fumarate Hydratase (FH), Malate dehydrogenase (MDH). The groups are represented by different colors as follows: mammals (brown); protozoan (blue); worms (yellow); bacteria (green); fungi (gold); plants (red); insects (light green); fish (cyan). Identified species: Hs (*Homo sapiens)*; Rn (*Rattus* novergicus); Tb (*trypanosoma* brucei); Tc (*Trypanosoma cruzi*); Tg (*Toxoplasma gondii*); Pf (*Plasmodium falciparum*); Te (*Trypanosoma evansi*); Gl (*Giardia lamblia*); Hm (*Haloferax mediterranei*); Bs (*Bacillus subtilis*); Ec (*Escherichia coli*); Mt (*Mycobacterium tuberculosis*); Pa (*Pseudomonas aeruginosa*); Tt (*Thermus thermophilus*); Vc (*Vibrio colerae*); Afl (*Aspergillus flavus*); Afu (*Aspergillus fumigatus*); Bb (*Beauveria bassiana*); Bc (*Botrytis cinerea*); Ca (*Candida albicans*); Cn (*Cryptococcus neoformans*); Fg (*Fusarium graminearium*); Hc (*Histoplasma capsulatum*); Mo (*Magnaporthe oryzae*); Ps (*Phytophthora sojae*); Sc (*Saccharomyces cerevisiae*); Tr (*Trichophyton rubrum*); Yl (*Yarrowia lipolytica*); At (*Arabdopsis thaliana*); Os (*Oryza sativa*); Ta (*Triticum aestivum*); Bd (*Brachypodium distachyon*); Vv (*Vitis vinifera*); Sj (*Schistosoma japonicum*); Ts (*Trichinella spiralis*); Bm (*Bombyx mori*); Dm (*Drosophila melanogaster*); Dr (*Danio rerio*).


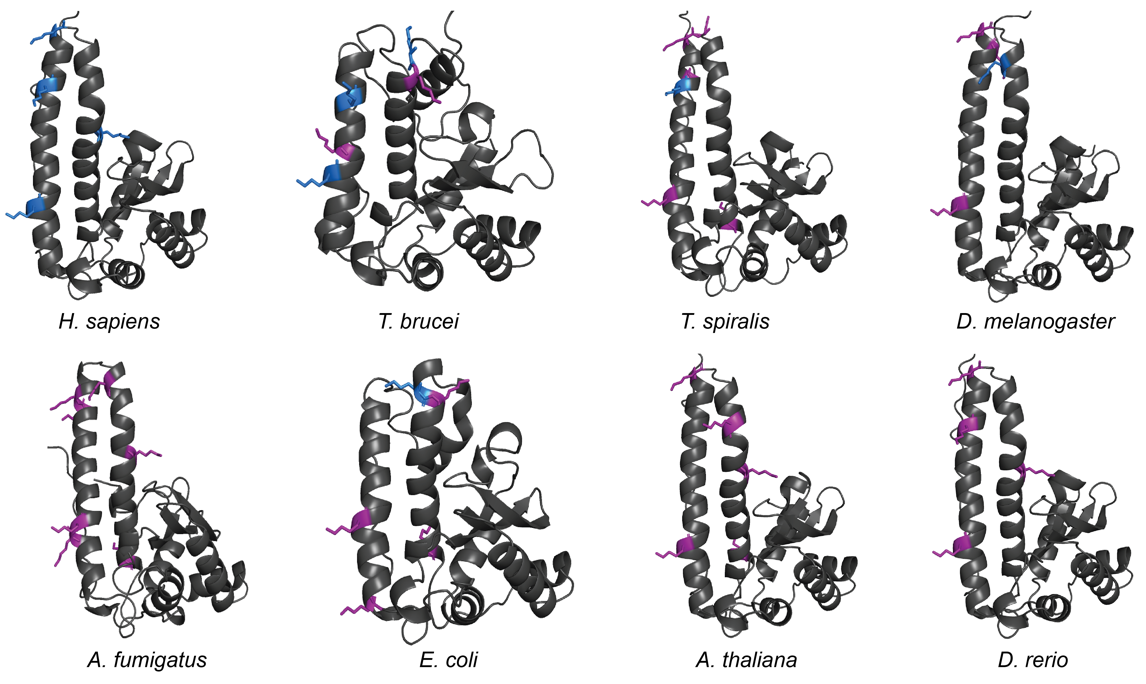


**Figure S9. Lysine acetylation of the superoxide dismutase A across different groups.** Highlighted within these structures are the lysine (K) residues identified as acetylated in their corresponding acetylomes (blue). Notably, several of these acetylated residues are located within the enzyme's funnel region, which plays a crucial role in directing substrates towards the catalytic site. Blue: lysine residues found acetylated. Purple: lysine residues not yet detected acetylated.
